# Supplementary material for: The lncRNA Neat1 promotes activation of inflammasomes in macrophages
Source: Nat Commun. 2019 Apr 2;10:1495. doi: 10.1038/s41467-019-09482-6 (PMC6445148; doi:10.1038/s41467-019-09482-6)
Supplement: Supplementary file 1 — Supplementary Information [file 41467_2019_9482_MOESM1_ESM.pdf]

Supplementary Information

The lncRNA *Neat1* promotes activation of inflammasomes in macrophages

Zhang et al.

Supplementary Figure 1

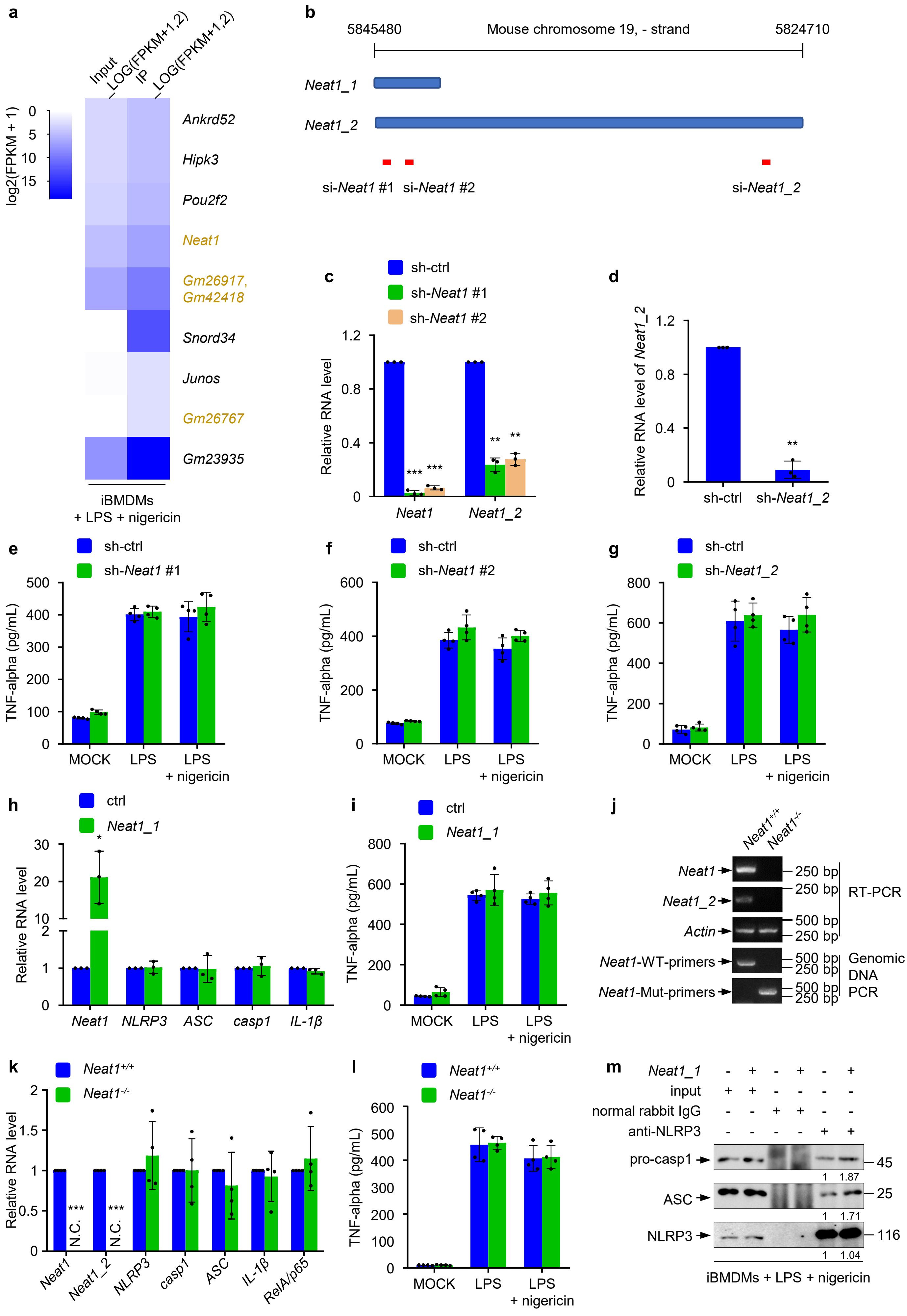

Supplementary Figure 1. Related to Figure 1.
(**a**) Heatmap of long noncoding RNA genes enriched by NLRP3 CLIP in iBMDMs that were primed with LPS and then stimulated with nigericin.
(**b**) Schematic illustration of *Neat1* genomic localization, isoforms, and shRNA/siRNA targeting sites.
(**c**, **d**) iBMDM cells were infected with lentiviruses expressing control shRNA, shRNAs targeted both isoforms of *Neat1* (sh-*Neat1* #1 and #2, **c**) or shRNA specifically targeted *Neat1_2* (sh-*Neat1_2*, **d**). Ninety-six hours after infection, efficiency of *Neat1* (**c,** left) or *Neat1_2* (**c**, right and **d**) knockdown was analyzed by real-time RT-PCR.
(**e-g**) Control iBMDMs, iBMDMs devoid of *Neat1* by shRNA #1 (**e**) or shRNA #2 (**f**), and iBMDMs devoid of *Neat1_2* (**g**) were treated with or without LPS and/or nigericin as indicated. Cell culture supernatants were analyzed by ELISA for TNF-alpha secretion.
(**h**) iBMDM cells were treated with lentiviruses expressing control RNA or *Neat1_1*. Ninety-six hours after injection, total RNA was analyzed by real-time RT-PCR.
(**i**) Control and *Neat1_1*-overexpressing iBMDMs were primed with LPS, and then stimulated with nigericin. Cell culture supernatants were analyzed by ELISA for TNF-alpha secretion.
(**j**) Mouse-tail genomic DNA and cDNA from total RNA of BMDMs were analyzed by PCR with indicated primers to identify *Neat1* wild-type ($Neat1^{+/+}$) and knockout ($Neat1^{-/-}$) mice.
(**k**) Total RNA of $Neat1^{+/+}$ or $Neat1^{-/-}$ BMDMs was subjected to be analyzed by real-time RT-PCR with indicated primers.
(**l**) BMDMs from $Neat1^{+/+}$ or $Neat1^{-/-}$ mice were primed with LPS, and then treated with nigericin. Culture supernatants were analyzed by ELISA for TNF-alpha secretion.
(**m**) Control iBMDMs and iBMDMs expressing ectopic *Neat1_1* were stimulated with LPS and nigericin. Cell lysates were immunoprecipitated with normal rabbit IgG or NLRP3 antibody. The samples were analyzed by Western blotting.
In **c**, **d** and **h**, data shown are mean ± SD (n = 3). In **e**, **f**, **g**, **i**, **k** and **l,** data shown are mean ± SD (n = 4). $*P < 0.05$, $**P < 0.01$, $***P < 0.001$, two-tailed t-test. Source data are provided as a Source Data file.

Supplementary Figure 2

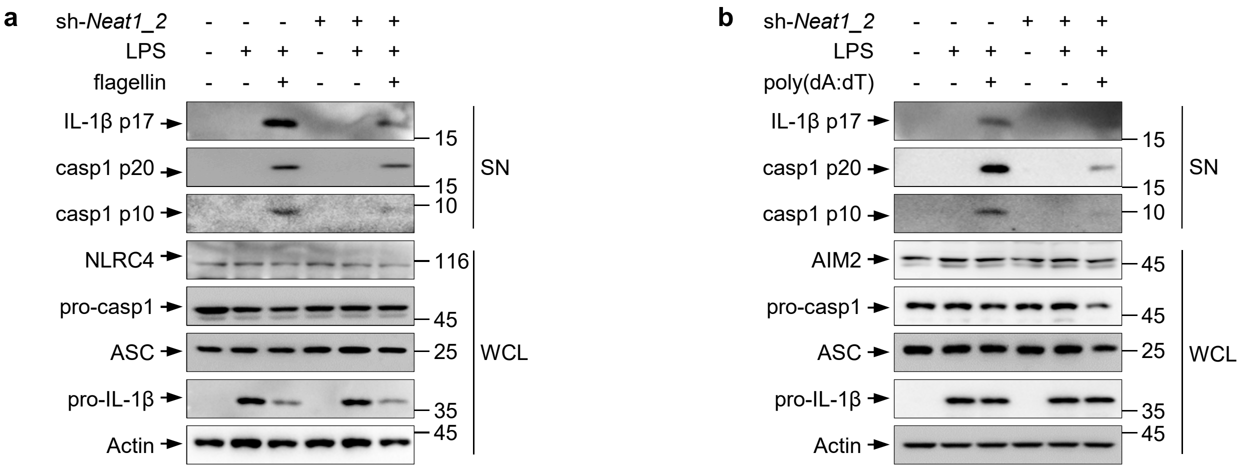

Supplementary Figure 2. Related to Figure 2.
(**a**, **b**) Control iBMDMs and iBMDMs devoid of *Neat1_2* were primed with LPS, and then stimulated with flagellin (**a**) or poly(dA:dT) (**b**). WCL and SN were analyzed by Western blotting.
Source data are provided as a Source Data file.

Supplementary Figure 3

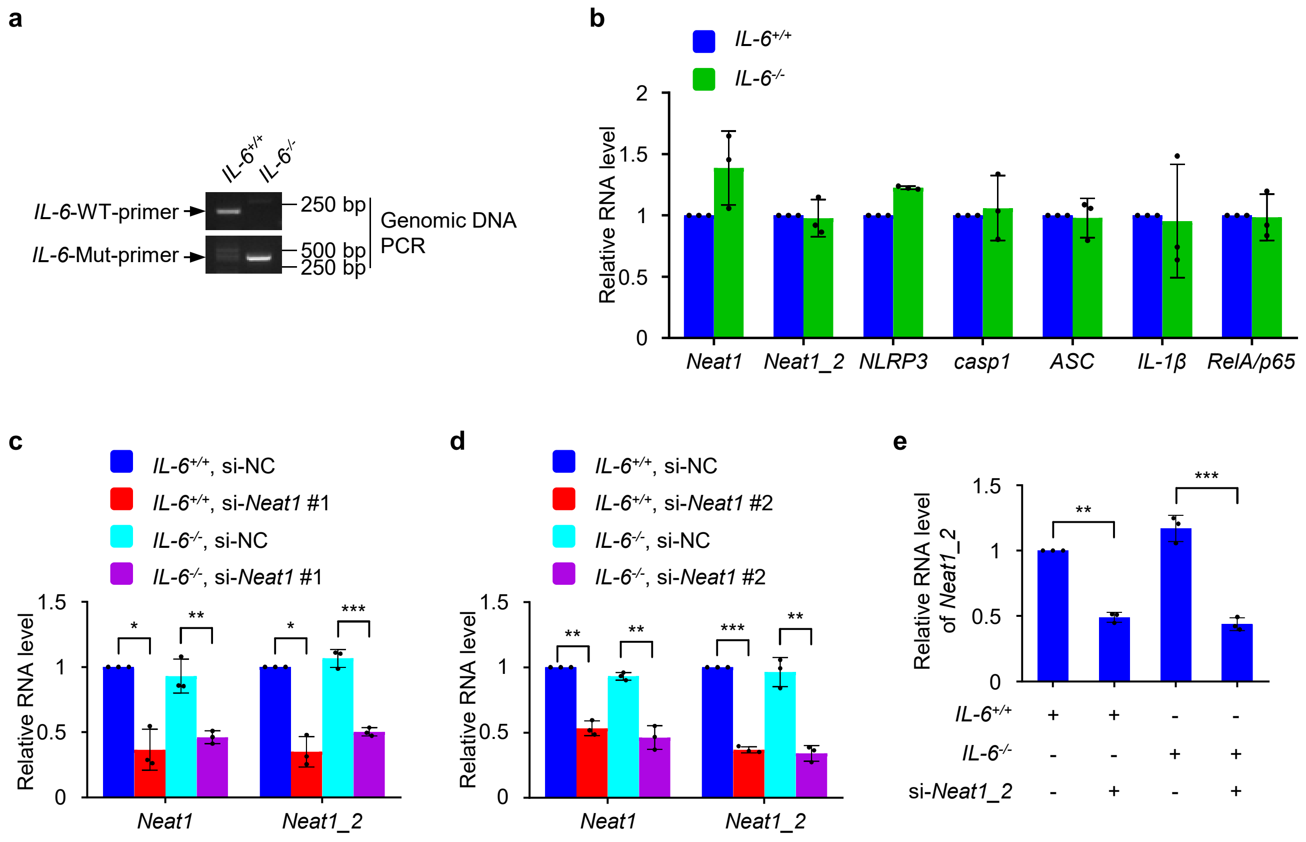

Supplementary Figure 3. Related to Figure 3.
(**a**) Mouse-tail genomic DNA was analyzed by PCR to identify *IL-6*$^{+/+}$ and *IL-6*$^{-/-}$ mice.
(**b**) Total RNA of *IL-6*$^{+/+}$ BMDMs or *IL-6*$^{-/-}$ BMDMs was analyzed by real-time RT-PCR with indicated primers.
(**c-e**) *IL-6*$^{+/+}$ or *IL-6*$^{-/-}$ BMDMs were transfected with control siRNA (**c-e**), *Neat1* siRNA #1 (**c**), *Neat1* siRNA #2 (**d**) or *Neat1_2* siRNA (**e**). Twenty-four hours later, cells were stimulated with LPS and nigericin. Total RNA was analyzed by real-time RT-PCR with indicated primers.
In **b**, **c**, **d** and **e**, data shown are mean ± SD (n = 3). **P* < 0.05, ***P* < 0.01, ****P* < 0.001, two-tailed t-test. Source data are provided as a Source Data file.

Supplementary Figure 4

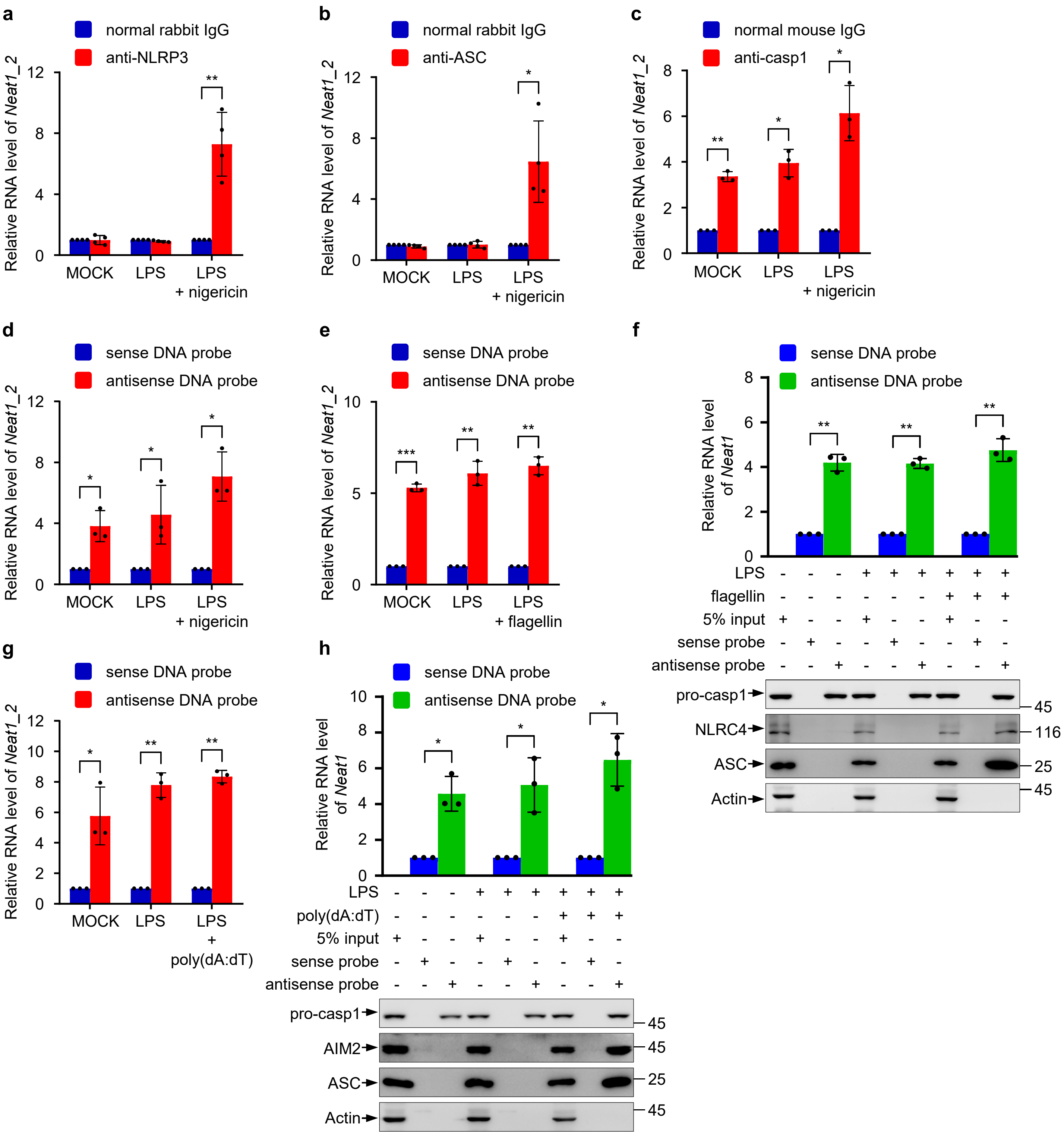

Supplementary Figure 4. Related to Figure 4.
(**a-c**) Cell lysates of normal iBMDMs, LPS-primed iBMDMs or LPS-nigericin-co-stimulated iBMDMs were incubated with normal rabbit IgG (**a-c**), NLRP3 antibody (**a**), ASC antibody (**b**) or caspase-1 antibody (**c**) for RIP. The immunoprecipitates were analyzed by real-time RT-PCR to exam the enrichment of *Neat1_2*.
(**d**, **e** and **g**) iBMDMs were untreated (**d**, **e** and **g**), primed with LPS (**d**, **e** and **g**), primed with LPS and then treated with nigericin (**d**), flagellin (**e**) or poly(dA:dT) (**g**). Cell lysates were incubated with biotin-labeled *Neat1* sense or antisense DNA probes immobilized on beads. The precipitated samples were analyzed by real-time RT-PCR for *Neat1_2* contents.
(**f**, **h**) Cell lysates of normal (**f**, **h**), LPS-primed (**f**, **h**), flagellin-activated (**f**) or poly(dA:dT)-activated (**h**) iBMDMs were incubated with biotin-labeled *Neat1* sense or antisense DNA probes immobilized on beads. The precipitated samples were analyzed by real-time RT-PCR for *Neat1* contents, and by Western blotting for indicated protein levels.
In **a** and **b**, data shown are mean ± SD (n = 4). In **c**, **d**, **e**, **f**, **g** and **h**, data shown are mean ± SD (n = 3). *$P < 0.05$, **$P < 0.01$, ***$P < 0.001$, two-tailed t-test. Source data are provided as a Source Data file.

Supplementary Figure 5

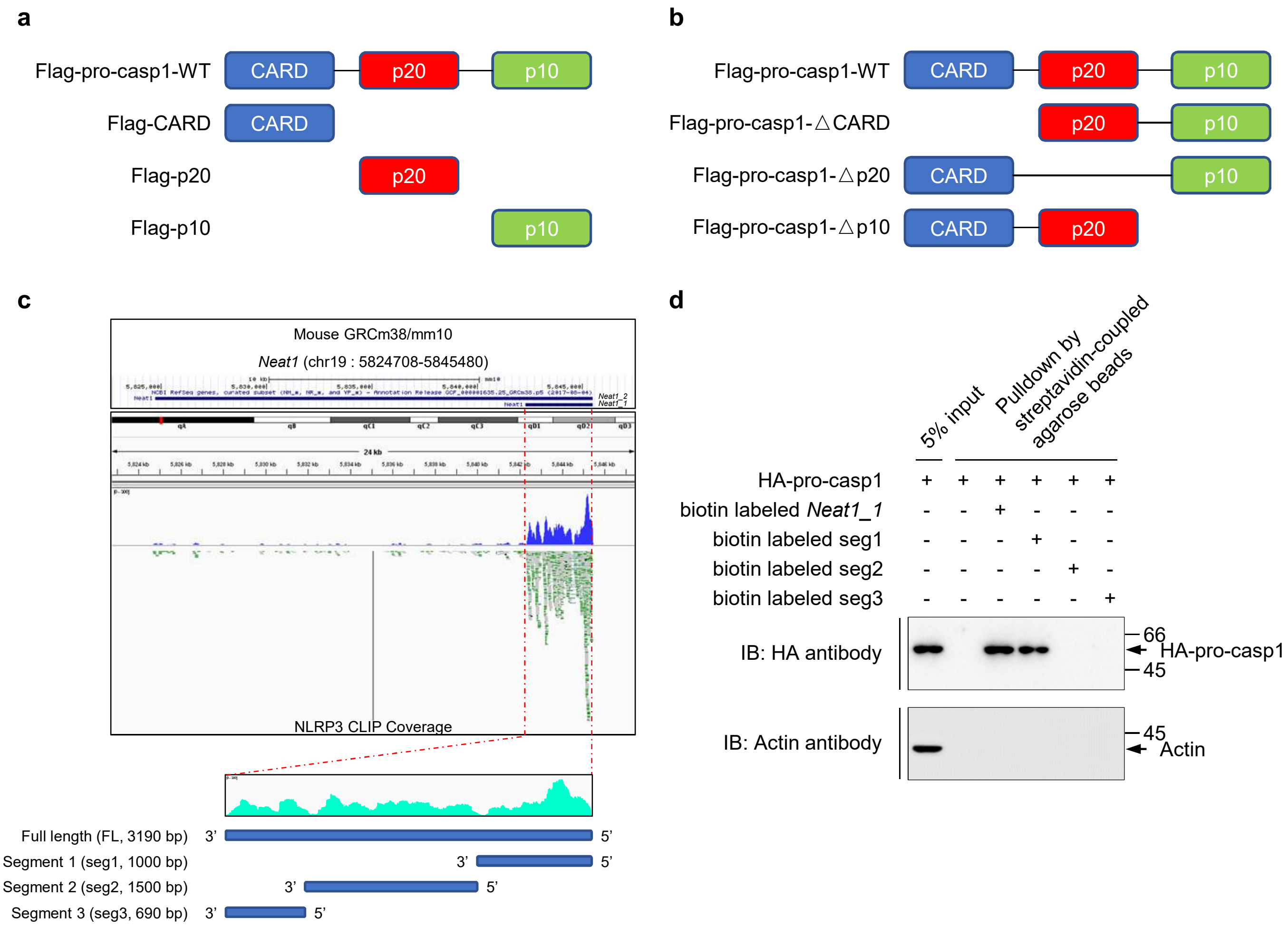

Supplementary Figure 5. Related to Figure 4.
(**a**, **b**) Schematic illustration of Flag-tagged pro-caspase-1 and its deletion mutants.
(**c**) Visualization analysis of the NLRP3 CLIP-seq peaks along *Neat1* was performed by Integrative Genomics Viewer (IGV). The predicted enriched RNA sequence was divided into three segments according to the distribution of the peaks as indicated in schematic illustration.
(**d**) *In vitro* transcribed, biotin labeled *Neat1_1* segments were separately incubated with lysates extracted from 293T cells expressing HA-tagged pro-caspase-1 for 3 hours. Cell lysates incubated with no RNA were used as negative controls. The input and biotin pull-down samples were then analyzed by Western blotting.
Source data are provided as a Source Data file.

Supplementary Figure 6

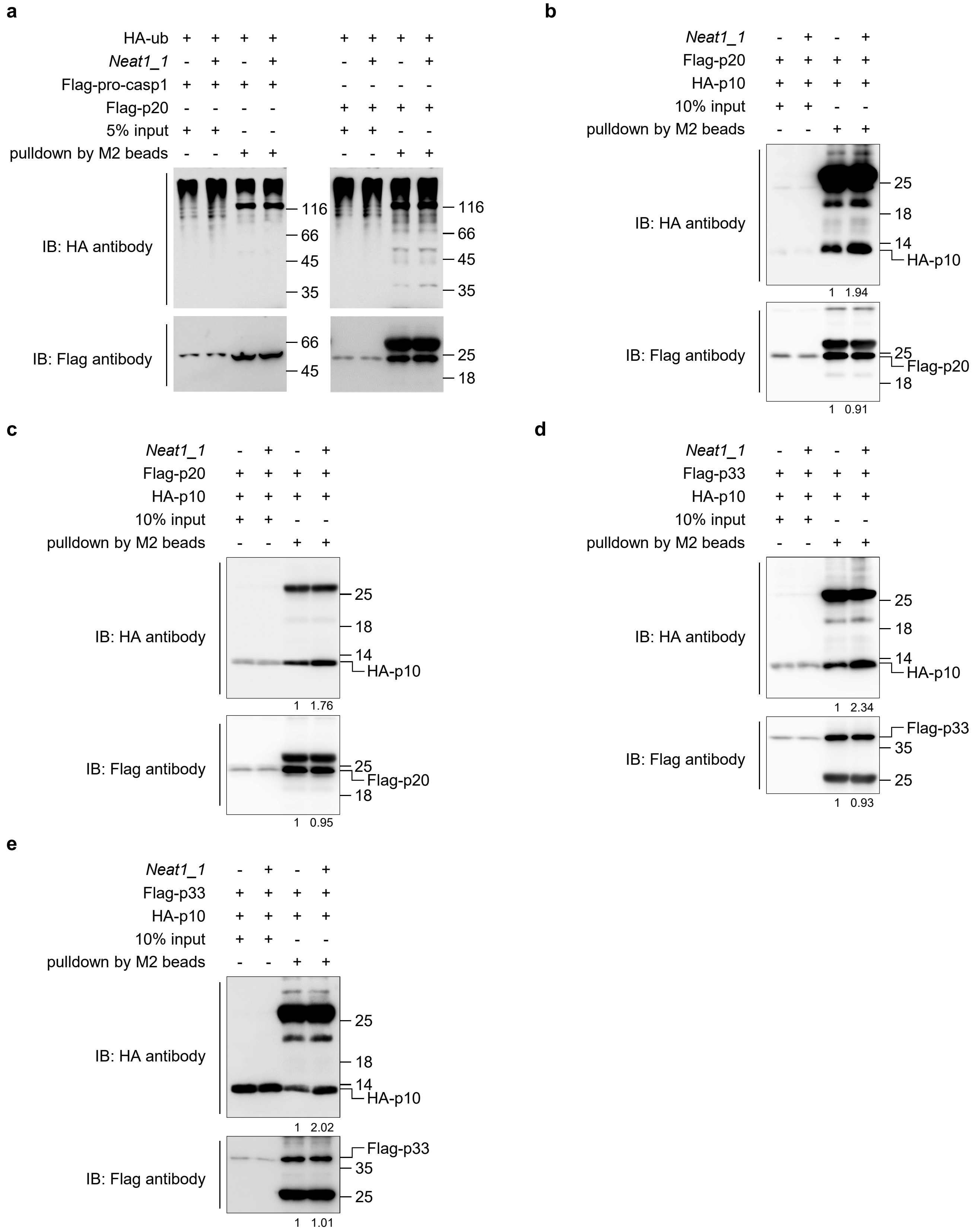

Supplementary Figure 6. Related to Figure 5.
(**a**) Control or *Neat1_1* overexpressing iBMDMs were co-transfected with HA-ub and Flag-pro-caspase-1/Flag-p20. Twenty-four hours later, cells were treated by MG132 for 6 hours. Cell lysates were incubated with M2 beads. Input and pull-down samples were subjected to Western blotting to analyze the ubiquitination level of Flag-pro-caspase-1 and Flag-p20.
(**b**-**e**) 293T cells were infected with lentiviruses expressing control RNA or *Neat1_1*. Ninety-six hours after injection, cells were co-transfected with Flag-p20 and HA-p10 (**b**, **c**), or Flag-p33 and HA-p10 (**d**, **e**). Twenty-four hours later, cell lysates were incubated with M2 beads. Input and pull-down samples were analyzed by Western blotting. The result are representative of three independent experiments.
Source data are provided as a Source Data file.

Supplementary Figure 7

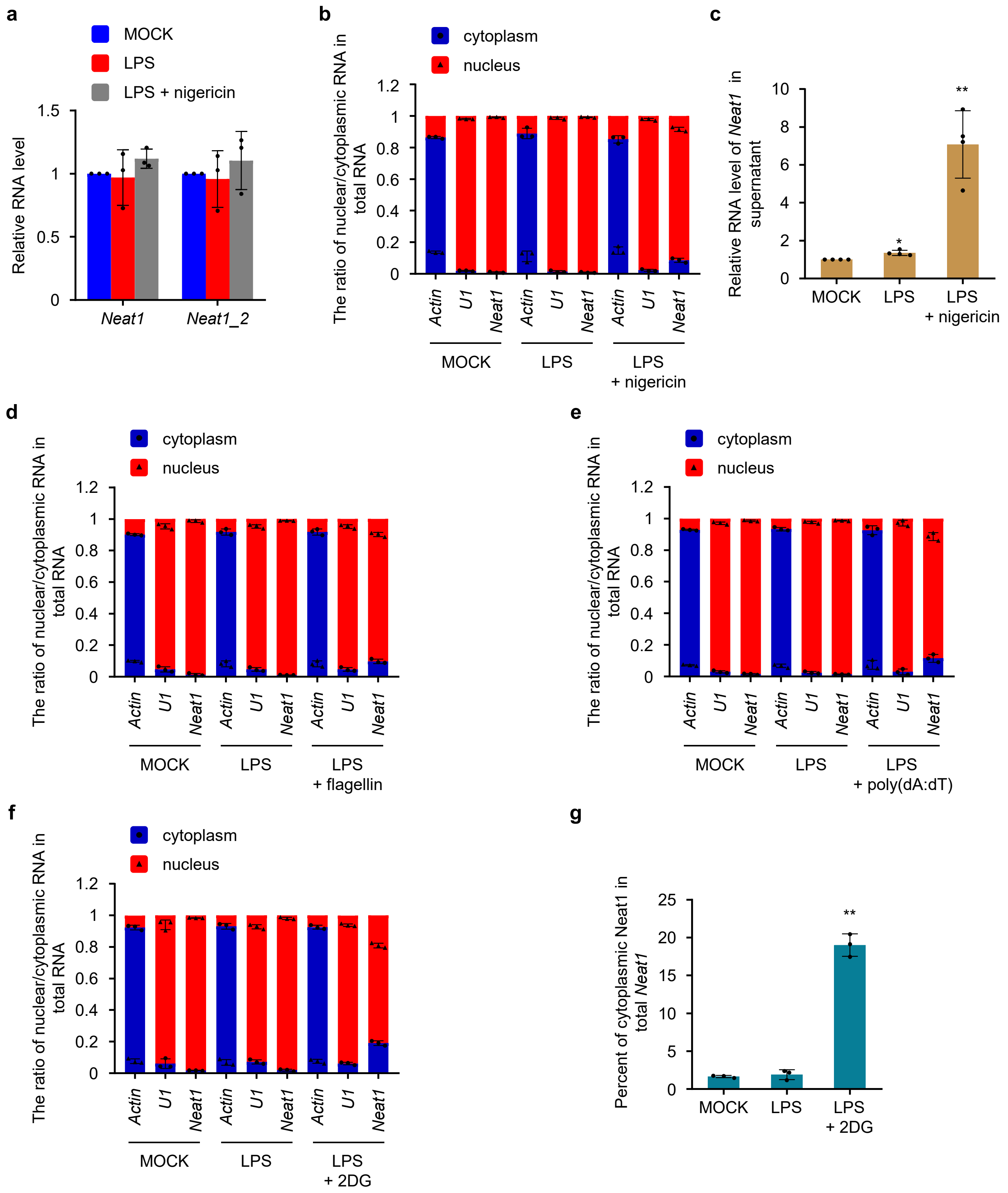

Supplementary Figure 7. Related to Figure 7.

(**a**) Total RNA of untreated, LPS-primed or LPS-nigericin-co-stimulated iBMDMs were analyzed by real-time RT-PCR.

(**b**) Untreated iBMDMs, LPS-primed iBMDMs or LPS-nigericin-co-stimulated iBMDMs were subjected to fractionation into cytoplasmic and nuclear extracts. Cytoplasmic and nuclear RNAs were analyzed for the indicated RNA expression by real-time RT–PCR. *Actin* and *U1* were used as markers for cytoplasmic and nuclear fractions, respectively.

(**c**) RNA from cell culture supernatant of untreated, LPS-primed or LPS-nigericin-co-stimulated iBMDMs was analyze for *Neat1* level by real-time RT–PCR.

(**d-f**) Untreated iBMDMs (**d-f**), LPS-primed iBMDMs (**d-f**), flagellin-activated iBMDMs (**d**), poly(dA:dT)-activated iBMDMs (**e**) or 2DG-activated iBMDMs (**f**) were subjected to fractionation into cytoplasmic and nuclear extracts. Cytoplasmic and nuclear RNAs were analyzed for the indicated RNA expression by real-time RT–PCR. *Actin* and *U1* were used as markers for cytoplasmic and nuclear fractions, respectively.

(**g**) Untreated iBMDMs, LPS-primed iBMDMs or LPS-2DG-co-stimulated iBMDMs were analyzed for cytoplasm *Neat1* levels.

In **a**, **b**, **d**, **e**, **f**, **g** and **h**, data shown are mean ± SD (n = 3). In **c**, data shown are mean ± SD (n = 4). ***P* < 0.01, two-tailed t-test. Source data are provided as a Source Data file.

Supplementary Figure 8

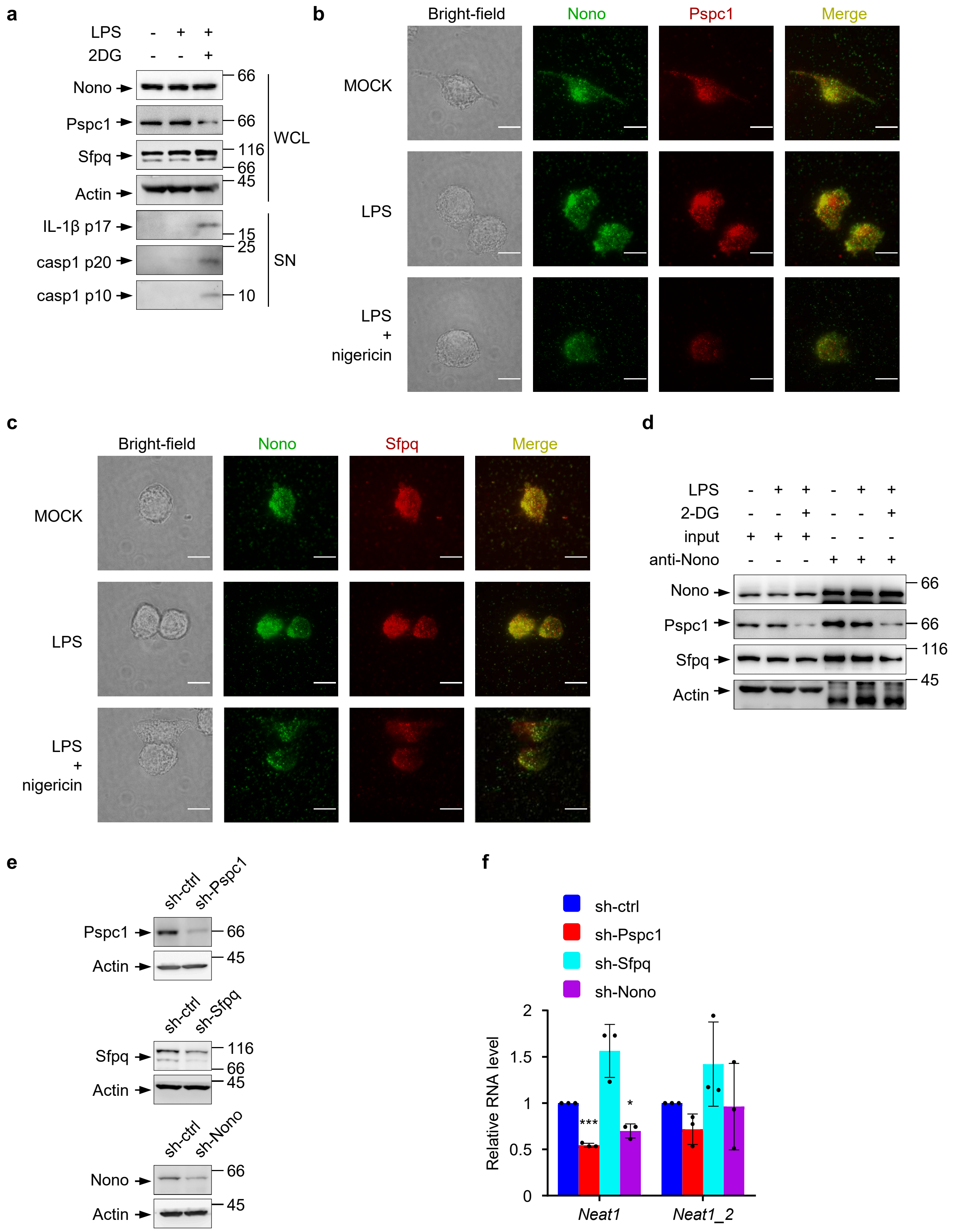

Supplementary Figure 8. Related to Figure 8.

(**a**) Cell lysates of untreated iBMDMs, LPS-primed iBMDMs or 2DG-activated iBMDMs were analyzed by Western blotting.

(**b**, **c**) Co-location of paraspeckle proteins, Nono (green; **b**, **c**) and Pspc1/Sfpq (red; **b**/**c**) were assayed by immunostaining. The location of paraspeckles (yellow) was obtained by merging two signals of Nono and Pspc1/Sfpq. Bright-field was used to observe the position of the nucleus.

(**d**) Cell lysates of untreated iBMDMs, LPS-primed iBMDMs or 2DG-activated iBMDMs were immunoprecipitated with Nono antibody. The immunoprecipitates were analyzed by Western blotting to detect the PPIs of paraspeckles.

(**e**) Western blotting was performed with cell lysates of control, Pspc1-silenced, Sfpq-silenced or Nono-silenced iBMDMs to detect the knockdown efficiency.

(**f**) Total RNA of control, Pspc1-silenced, Sfpq-silenced or Nono-silenced iBMDMs were subjected to analyzed by real-time RT-PCR to examine *Neat1* and *Neat1_2* expressional levels.

In **b** and **c**, the bar graph indicates 10 μm. In **f**, data shown are mean ± SD (n = 3). *$P < 0.05$, ***$P < 0.001$, two-tailed t-test. Source data are provided as a Source Data file.

Supplementary Figure 9

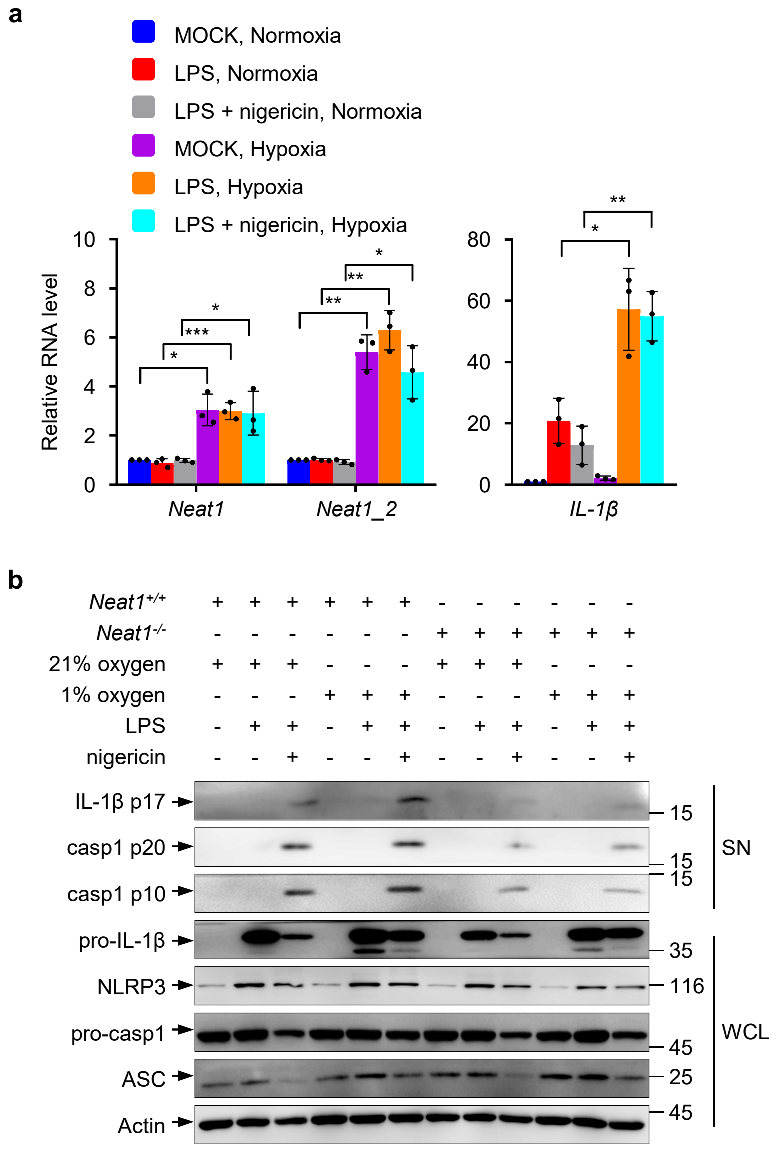

Supplementary Figure 9. Related to Figure 9.

(**a**) iBMDMs cultured under the condition of normoxia (21% oxygen) or hypoxia (1% oxygen) were untreated, primed with LPS, or co-stimulated with LPS and nigericin. Relative levels of *Neat1*, *Neat1_2*, and *IL-1β* (positive control) were analyzed by real-time RT-PCR.

(**b**) $Neat1^{+/+}$ or $Neat1^{-/-}$ BMDMs cultured under the condition of normoxia or hypoxia were primed with LPS, and then stimulated with nigericin. WCL and SN of above cells from different stages were subjected to Western blotting.

In **a**, data shown are mean ± SD (n = 3). **P* < 0.05, ***P* < 0.01, ****P* < 0.001, two-tailed t-test. Source data are provided as a Source Data file.

Supplementary Figure 9

a

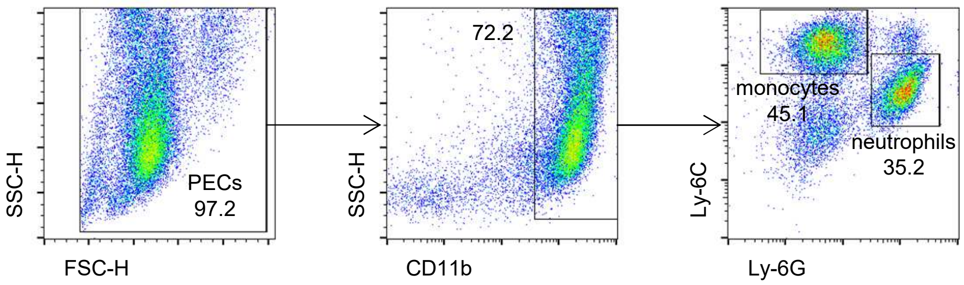

b

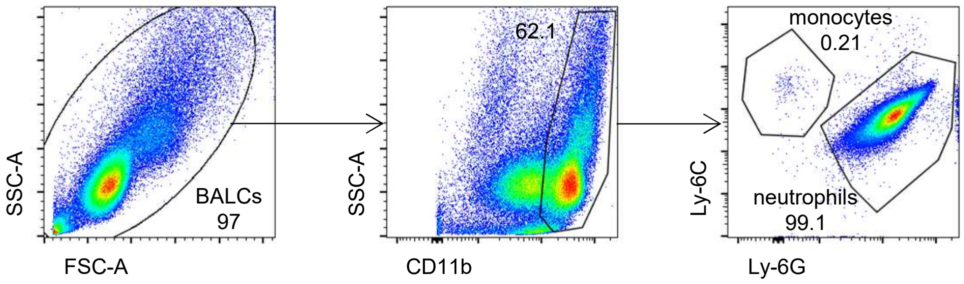

Supplementary Figure 10. Related to Figure 10.

(**a**, **b**) Gating strategy to determine the percentage of neutrophils (CD11b^{+}ly-6G^{+}) and inflammatory monocytes (CD11b^{+}ly-6C^{+}) in peritoneal exudate cells (**a**) and bronchoalveolar lavage (**b**), respectively.

Supplementary Figure 11

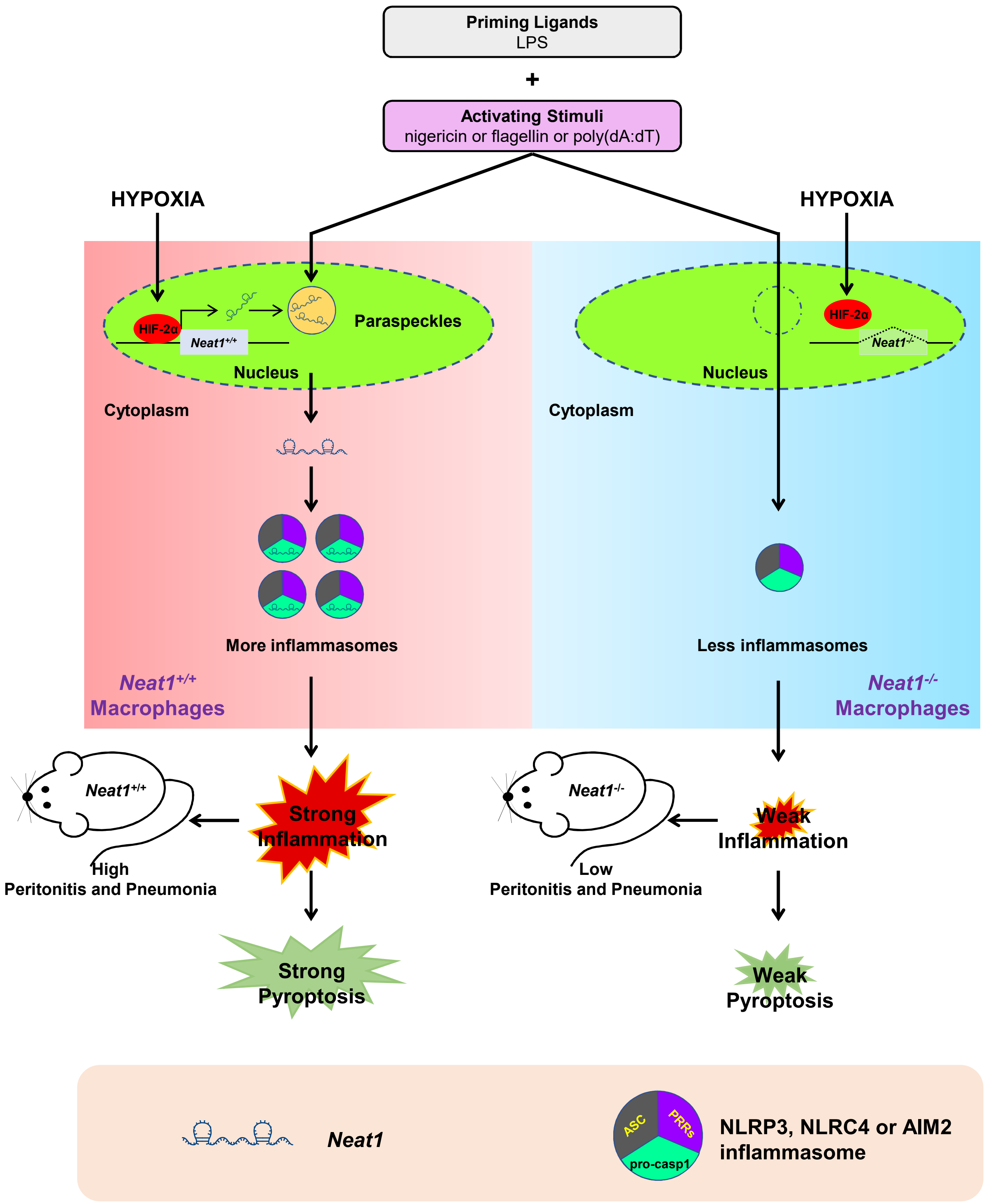

Supplementary Figure 11. Graphic abstract.
A model depicting the role of hypoxia-inducible *Neat1* in promoting the activation of various inflammasomes in macrophages, Alum-induced peritonitis and flagellin-induced pneumonia.

Supplementary Table 1. Oligomers used in this study

Name	Application	Sequence
Neat1-sgRNA #1	CRISPR	CUAUUUCUAGCUCUAAAACGUGGAGGAAUCGUUCCGUUUCCGGUGUUUCGUCCUUUCCA
Neat1-sgRNA #2	CRISPR	CUAUUUCUAGCUCUAAAACCUGUGAGCCGCAUGAUAGCCGGUGUUUCGUCCUUUCCA
Neat1-genotyping-WT-F	genotyping PCR	GGATATGCCCTTCATCCTGTGACGC
Neat1-genotyping-WT-R	genotyping PCR	TCCACACGTCCACTGTCCCAAAATC
Neat1-genotyping-KO-R	genotyping PCR	GTATCCATCAAACACCAGCAGCCTG
IL-6-genotyping-WT-F	genotyping PCR	TTCCATCCAGTTGCCTTCTTGG
IL-6-genotyping-WT-R	genotyping PCR	TTCTCATTTCCACGATTTCCCAG
IL-6-genotyping-KO-R	genotyping PCR	CCGGAGAACCTGCGTGCAATCC
Neat1-1-sense	lncRNA pull down	(biotin-)TGACAAGGAGGGCTCGCTCTT
Neat1-1-antisense	lncRNA pull down	(biotin-)AAGAGCGAGCCCTCCTTGTCA
Neat1-2-sense	lncRNA pull down	(biotin-)GCACAAGTTTCACAGGCCTAC
Neat1-2-antisense	lncRNA pull down	(biotin-)GTAGGCCTGTGAAACTTGTGC
Neat1-3-sense	lncRNA pull down	(biotin-)GACACCCTGACTGGGCGGGGA
Neat1-3-antisense	lncRNA pull down	(biotin-)TCCCCGCCCAGTCAGGGTGTC
Neat1-4-sense	lncRNA pull down	(biotin-)CACTATAGTGTTCACCATGGC
Neat1-4-antisense	lncRNA pull down	(biotin-)GCCATGGTGAACACTATAGTG
Neat1-5-sense	lncRNA pull down	(biotin-)TTCTGGACTAAAAGGGATCCG
Neat1-5-antisense	lncRNA pull down	(biotin-)CGGATCCCTTTTAGTCCAGAA
Neat1-6-sense	lncRNA pull down	(biotin-)GGTTGTCACTACCCTGACCTA
Neat1-6-antisense	lncRNA pull down	(biotin-)TAGGTCAGGGTAGTGACAACC
T7-*Neat1_1*-F	template amplification for *in vitro*-synthesized biotin-labeled RNA	TAATACGACTCACTATAGGGGAGTTAGTGACAAGGAGGGCTCGC
Neat1_1-R	template amplification for *in vitro*-synthesized biotin-labeled RNA	TTCAATCTCAAACCTTTATTTTGCTGTAAAGG
Neat1-FISH-probe	RNA-FISH	UGUUGCCACUCAACCACCCAGUAUCAAAUCCACACCCUGGGAGAGACCAUUCCCAGGGACCACUUCGGGCCCACGGAUCCCUUUUAGUCCAGAAGACGUCCCAAAGCCCAAAUUAGAUACGGCAUCUACCAAGUCGGCAGAAUUUGUGGCUAACUUGCGCCUUCCCACUGUUAAACCACAAACCCUACCCAUUUUACAACCAGCUCCAUCUAAGGAAGAUUCCUCCACACCAGUCAUUUACAACCCAACAGCUUUCCCCAACACCCACAAGUUUUCAGUUAAGAAUCCCUCUGACCAAUGCAGAGGCAGGCAGGCUGCAGAAGCAGGGAAGGUGGGCGAGGUCACUGGGGUCCCGAUCUACAGUGCAGCACGCUGCGCAGACGUUAAAGUGUCCU

		ACACCUUACGCAAUCUUCUCGAGGCCAAGUUCGCAGCACA GAACUACAAAGGCUGGAUGGAGGCUUGUUUAGAAGAUGC AGCAGUCGAACGCCUCUACCCCCAUG
qrt-*Actin*-F	qRT-PCR	GACCTGACTGACTACCTCATGAAGAT
qrt-*Actin*-R	qRT-PCR	GTCACACTTCATGATGGAGTTGAAGG
qrt-*U1*-F	qRT-PCR	GGCGAGGCTTATCCATTG
qrt-*U1*-R	qRT-PCR	CCCACTACCACAAATTATGC
qrt-*IL-1β*-F	qRT-PCR	TTCCTTGTGCAAGTGTCTGAAG
qrt-*IL-1β*-R	qRT-PCR	CACTGTCAAAAGGTGGCATTT
qrt-*NLRP3*-F	qRT-PCR	AGAAGAGACCACGGCAGAAG
qrt-*NLRP3*-R	qRT-PCR	CCTTGGACCAGGTTCAGTGT
qrt-*ASC*-F	qRT-PCR	GAGCAGCTGCAAACGACTAA
qrt-*ASC*-R	qRT-PCR	GCTGGTCCACAAAGTGTCCT
qrt-*caspase-1*-F	qRT-PCR	TCAGCTCCATCAGCTGAAAC
qrt-*caspase-1*-R	qRT-PCR	TGGAAATGTGCCATCTTCTTT
qrt-*p65*-F	qRT-PCR	TGTGGAGATCATCGAACAGCCG
qrt-*p65*-R	qRT-PCR	TTCCTGGTCCTGTGTAGCCATTGAT
qrt-*Neat1*-F	qRT-PCR	GGCACAAGTTTCACAGGCCTACATGGG
qrt-*Neat1*-R	qRT-PCR	GCCAGAGCTGTCCGCCCAGCGAAG
qrt-*Neat1_2*-F	qRT-PCR	GGAGCCAACCTGCCCTGAAT
qrt-*Neat1_2*-R	qRT-PCR	CCACAGGCTACCCTCTGCTC
sh-*Neat1* #1-F	plasmid construction	ccggCAGGACTAGGTGCGTAGTGctcgagCACTACGCACCTAGTC CTGtttttg
sh-*Neat1* #1-R	plasmid construction	aattcaaaaaCAGGACTAGGTGCGTAGTGctcgagCACTACGCACCT AGTCCTG
sh-*Neat1* #2-F	plasmid construction	ccggGAGAAGATTGCGTAAGGTGTAggatccTACACCTTACGCAA TCTTCTCtttttg
sh-*Neat1* #2-R	plasmid construction	aattcaaaaaGAGAAGATTGCGTAAGGTGTAggatccTACACCTTAC GCAATCTTCTC
sh-*Neat1_2*-F	plasmid construction	ccggGAGTTACCATCCCGTCCTCTAggatccTAGAGGACGGGATG GTAACTCtttttg
sh-*Neat1_2*-R	plasmid construction	aattcaaaaaGAGTTACCATCCCGTCCTCTAggatccTAGAGGACGG GATGGTAACTC
si-*Neat1* #1	siRNA (sense, 5’-3’)	CAGGACTAGGTGCGTAGTGTT
si-*Neat1* #2	siRNA (sense, 5’-3’)	GAGAAGATTGCGTAAGGTGTATT
si-*Neat1_2*	siRNA (sense, 5’-3’)	GAGTTACCATCCCGTCCTCTATT
