# Supplementary material for: The lncRNA Neat1 promotes activation of inflammasomes in macrophages
Source: Nat Commun. 2019 Apr 2;10:1495. doi: 10.1038/s41467-019-09482-6 (PMC6445148; doi:10.1038/s41467-019-09482-6)
Supplement: Supplementary file 3 — Reporting Summary [file 41467_2019_9482_MOESM3_ESM.pdf]

nature research

Corresponding author(s): Mian Wu

Last updated by author(s): Mar 13, 2019

Reporting Summary

Nature Research wishes to improve the reproducibility of the work that we publish. This form provides structure for consistency and transparency in reporting. For further information on Nature Research policies, see Authors & Referees and the Editorial Policy Checklist.

Statistics

For all statistical analyses, confirm that the following items are present in the figure legend, table legend, main text, or Methods section.

n/a	Confirmed	
☐	☒	The exact sample size (*n*) for each experimental group/condition, given as a discrete number and unit of measurement
☐	☒	A statement on whether measurements were taken from distinct samples or whether the same sample was measured repeatedly
☐	☒	The statistical test(s) used AND whether they are one- or two-sided<br>*Only common tests should be described solely by name; describe more complex techniques in the Methods section.*
☒	☐	A description of all covariates tested
☒	☐	A description of any assumptions or corrections, such as tests of normality and adjustment for multiple comparisons
☐	☒	A full description of the statistical parameters including central tendency (e.g. means) or other basic estimates (e.g. regression coefficient) AND variation (e.g. standard deviation) or associated estimates of uncertainty (e.g. confidence intervals)
☐	☒	For null hypothesis testing, the test statistic (e.g. *F*, *t*, *r*) with confidence intervals, effect sizes, degrees of freedom and *P* value noted<br>*Give P values as exact values whenever suitable.*
☒	☐	For Bayesian analysis, information on the choice of priors and Markov chain Monte Carlo settings
☒	☐	For hierarchical and complex designs, identification of the appropriate level for tests and full reporting of outcomes
☒	☐	Estimates of effect sizes (e.g. Cohen's *d*, Pearson's *r*), indicating how they were calculated

Our web collection on statistics for biologists contains articles on many of the points above.

Software and code

Policy information about availability of computer code

Data collection	MxPro Application; BioSens Gel Imaging System; SageCapture; Image Reader LAS-4000; DPController; CellQuest Pro; LAS X.
Data analysis	Microsoft Office; GraphPad Prism 8; Origin 8; IGV; Image J; FlowJo7.6; R x64 3.4.0.

For manuscripts utilizing custom algorithms or software that are central to the research but not yet described in published literature, software must be made available to editors/reviewers. We strongly encourage code deposition in a community repository (e.g. GitHub). See the Nature Research guidelines for submitting code & software for further information.

Data

Policy information about availability of data

All manuscripts must include a data availability statement. This statement should provide the following information, where applicable:

- Accession codes, unique identifiers, or web links for publicly available datasets
- A list of figures that have associated raw data
- A description of any restrictions on data availability

We have uploaded the raw data and processed data of NLRP3 CLIP-seq to Gene Expression Omnibus (GEO).The accession number is GSE118722.

Field-specific reporting

Please select the one below that is the best fit for your research. If you are not sure, read the appropriate sections before making your selection.

☒ Life sciences ☐ Behavioural & social sciences ☐ Ecological, evolutionary & environmental sciences

For a reference copy of the document with all sections, see nature.com/documents/nr-reporting-summary-flat.pdf

Life sciences study design

All studies must disclose on these points even when the disclosure is negative.

Sample size	No statistical method was used to predetermine sample size; sample sizes were chosen according to experimental standard procedures for the respective assays.
Data exclusions	No data were excluded for this study.
Replication	The shown experiments could successfully and reliably be replicated and reproduced.
Randomization	We have used unbiased approach in choosing mice for in vivo peritonitis assays. Mice were used in the experiment at random.
Blinding	During testing the PECs, the BALCs and the concentrations of mouse IL-1β, the experimentalists were blinded to the information of mice.

Reporting for specific materials, systems and methods

We require information from authors about some types of materials, experimental systems and methods used in many studies. Here, indicate whether each material, system or method listed is relevant to your study. If you are not sure if a list item applies to your research, read the appropriate section before selecting a response.

Materials & experimental systems

n/a	Involved in the study
☐	☒ Antibodies
☐	☒ Eukaryotic cell lines
☒	☐ Palaeontology
☐	☒ Animals and other organisms
☒	☐ Human research participants
☒	☐ Clinical data

Methods

n/a	Involved in the study
☒	☐ ChIP-seq
☐	☒ Flow cytometry
☒	☐ MRI-based neuroimaging

Antibodies

Antibodies used	Antibodies used for Western bloting included anti-IL-1β (R&D Systems, 1:1000, AF-401-NA), anti-HIF-2α (R&D Systems, 1:1000, AF2997), anti-NLRP3 (Adipogen, 1:1000, AG-20B-0014), anti-ASC (Adipogen, 1:1000, AG-25B-0006), anti-caspase-1 (p20) (Adipogen, 1:1000, AG-20B-0042), anti-caspase-1 (p10) (Adipogen, 1:1000, AG-20B-0044), anti-p65 (Cell Signaling Technology, 1:1000, 8242), anti-HIF-1α (Cell Signaling Technology, 1:1000, 14179), anti-AIM2 (Cell Signaling Technology, 1:1000, 13095), anti-IL-6 (Cell Signaling Technology, 1:1000, 12912), anti-Actin (CMC-TAG, 1:2000, AT0009), anti-FLAG (Sigma-Aldrich, 1:2000, F3165), anti-Pspc1 (Santa Cruz, 1:200, sc-374181), anti-Sfpq (Santa Cruz, 1:200, sc-271796), anti-Nono (ABclonal, 1:500, A5282), anti-NLRC4 (ABclonal, 1:500, A7382), anti-histone H2A (abcam, 1:1000, ab177308), HRP-linked anti-rabbit IgG (Cell Signaling Technology, 1:2000, 7074), HRP-linked anti-mouse IgG (Cell Signaling Technology, 1:2000, 7076), HRP-linked anti-goat IgG (Santa Cruz, 1:1000, sc-2354). Antibodies used for immunoprecipitation included anti-NLRP3 (Cell Signaling Technology, 1:200, 15101), anti-caspase-1 (p20) (Adipogen, 1:200, AG-20B-0042), anti-ASC (Adipogen, 1:200, AG-25B-0006), anti-Nono (ABclonal, 1:50, A5282), normal rabbit IgG (Cell Signaling Technology, 1:200, 2729), normal mouse IgG (Santa Cruz, 1:80, sc-2025). Anti-ASC (Santa Cruz, 1:50, sc-22514), anti-Pspc1 (mouse IgG, Santa Cruz, 1:50, sc-374181), anti-Sfpq (mouse IgG, Santa Cruz, 1:50, sc-271796), anti-Sfpq (rabbit IgG, Proteintech, 1:50, 15585-1-AP), anti-Nono (rabbit IgG, ABclonal, 1:50, A5282), Alexa fluor 488 donkey anti-rabbit IgG(H+L) (Life Technologies, 1:1000, A21206), Alexa fluor 568 donkey anti-mouse IgG(H+L) (Life Technologies, 1:1000, A10037) and Alexa fluor 647 donkey anti-rabbit IgG(H+L) (Life Technologies, 1:1000, A31573) were used for immunofluorescence. PerCP-CyTM5.5 anti-mouse CD11b (BD Biosciences, 1:1000, 550993), APC anti-mouse Ly-6G (BD Biosciences, 1:1000, 560599) and PE anti-mouse Ly-6C (BD Biosciences, 1:1000, 560592) were used for flow cytometry.
Validation	All informations of antibodies used were available as shown in the first Paragraph of Experimental Procedures section.

Eukaryotic cell lines

Policy information about cell lines

Cell line source(s)	Information and references to the used cell lines are provided in the manuscript.
Authentication	All cells were tested by STR profiling with STR markers to authenticate the identity according to the method of ATCC and the published protocol (PMID: 23430347).
Mycoplasma contamination	All cells were tested for mycoplasma contamination by Cell Culture Contamination Detection Kit (ThermoFisher).
Commonly misidentified lines (See ICLAC register)	No commonly misidentified cell lines were used.

Animals and other organisms

Policy information about studies involving animals; ARRIVE guidelines recommended for reporting animal research

Laboratory animals	Neat1 wild-type mice and Neat1 knockout mice, males, 8 weeks old.
Wild animals	This study did not involve wild animals. samples collected from field.
Field-collected samples	This study did not involve samples collected from field.
Ethics oversight	All animal experiments were undertaken and conducted with approval from the Animal Research Ethics Committee of the University of Science and Technology of China (Permit Number: USTCACUC1801057).

Note that full information on the approval of the study protocol must also be provided in the manuscript.

Flow Cytometry

Plots

Confirm that:

- [x] The axis labels state the marker and fluorochrome used (e.g. CD4-FITC).
- [x] The axis scales are clearly visible. Include numbers along axes only for bottom left plot of group (a 'group' is an analysis of identical markers).
- [x] All plots are contour plots with outliers or pseudocolor plots.
- [x] A numerical value for number of cells or percentage (with statistics) is provided.

Methodology

Sample preparation	PECs and BALCs were obtained and blocked in PBS with 20% rat serum for 0.5 hours. After washing three times with PBS, cells were incubated with PerCP-CyTM5.5-mouse CD11b antibody, APC-mouse Ly-6G antibody and PE-mouse Ly-6C antibody for 1 hour. After washing three times with PBS, stained cells were analyzed on the BD FACSCalibur to detect the proportion of neutrophils or Ly-6C+ monocytes. The flow cytometry data were analyzed on FlowJo software with the gating strategies indicated in Supplementary Fig. 10.
Instrument	BD FACSCalibur
Software	CellQuest Pro
Cell population abundance	Neutrophils are APC/PerCP-CyTM5.5-double-positve. Monocytes were PE/PerCP-CyTM5.5-double-positve.
Gating strategy	Neutrophils were defined as APC/PerCP-CyTM5.5-double-positve. Monocytes were defined as PE/PerCP-CyTM5.5-double-positve. The gating strategies were shown in Supplementary Figure 10.

- [x] Tick this box to confirm that a figure exemplifying the gating strategy is provided in the Supplementary Information.
